# Design, Synthesis and Biological Evaluation of Multi-Target Anti-Cancer Agent PYR26

**DOI:** 10.3390/ijms24087131

**Published:** 2023-04-12

**Authors:** Sirong He, Peiting He, Haojing Wu, Yao Feng, Jiejin Situ, Yiling Chen, Junxi Du, Jin Qin, Pengcheng Lv, Kun Chen

**Affiliations:** The Joint Research Center of Guangzhou University and Keele University for Gene Interference and Application, School of Life Science, Guangzhou University, Guangzhou 510006, China

**Keywords:** liver cancer, anti-cancer agent, multiple targets, PYR26, proliferation inhibition, promote apoptosis

## Abstract

This study investigates the synthesis of a new compound, PYR26, and the multi-target mechanism of PYR26 inhibiting the proliferation of HepG2 human hepatocellular carcinoma cells. PYR26 significantly inhibits the growth of HepG2 cells (*p* < 0.0001) and this inhibition has a concentration effect. There was no significant change in ROS release from HepG2 cells after PYR26 treatment. The mRNA expressions of *CDK4*, *c-Met* and *Bak* genes in HepG2 cells were significantly inhibited (*p* < 0.05), while mRNA expression of pro-apoptotic factors such as caspase-3 and Cyt c was significantly increased (*p* < 0.01). The expression of PI3K, CDK4 and pERK proteins decreased. The expression level of caspase-3 protein was increased. PI3K is a kind of intracellular phosphatidylinositol kinase. PI3K signaling pathway is involved in signal transduction of a variety of growth factors, cytokines and extracellular matrix and plays an important role in preventing cell apoptosis, promoting cell survival and influencing cell glucose metabolism. CDK4 is a catalytic subunit of the protein kinase complex and is important for G1 phase progression of the cell cycle. PERK refers to phosphorylated activated ERK, which is translocated from cytoplasm to the nucleus after activation, and then participates in various biological reactions such as cell proliferation and differentiation, cell morphology maintenance, cytoskeleton construction, cell apoptosis and cell canceration. Compared with the model group and the positive control group, the tumor volume of the nude mice in the low-concentration PYR26 group, the medium-concentration group and the high-concentration group was smaller, and the organ volume was smaller than that in the model group and the positive control group. The tumor inhibition rates of low-concentration group PYR26, medium-concentration group and high-concentration group reached 50.46%, 80.66% and 74.59%, respectively. The results showed that PYR26 inhibited the proliferation of HepG2 cells and induced apoptosis of HepG2 cells by down-regulating *c-Met*, *CDK4* and *Bak*, up-regulating the mRNA expression of *caspase-3* and *Cyt c* genes, down-regulating PI3K, pERK and CDK4 proteins and up-regulating the protein level of caspase-3. In a certain range, with the increase in PYR26 concentration, the tumor growth was slower and the tumor volume was smaller. Preliminary results showed that PYR26 also had an inhibitory effect on the tumors of Hepa1-6 tumor-bearing mice. These results suggest that PYR26 has an inhibitory effect on the growth of liver cancer cells, therefore it has potential to be developed into a new anti-liver cancer drug.

## 1. Introduction

Liver cancer is a malignant tumor that occurs in the liver, which can be divided into two major categories, primary and secondary. Liver cancer is ranked 3rd among fatal cancer worldwide [1]. A study’s statistics show that the incidence rate of liver cancer in China ranked 5th among malignant tumors and 2nd in mortality in 2020 [2], seriously threatening the lives and health of people around the world. In liver cancer patients, cancer cells increase, and the liver gradually loses its normal function [3]. In traditional treatment protocols, chemotherapy, radiotherapy and surgical resection are the main ways to treat tumor diseases [4]. However, apoptosis is not the only means to kill and eradicate cancer cells, and chemotherapeutic drugs often suffer from poor water solubility and low tumor targeting ability [5], with limited effect, which will also seriously affect the quality of life of patients and therefore greatly limit their clinical applications [6]. Molecular testing brings new therapeutic breakthroughs for tumors, including specific gene mutations, amplification or fusion, epigenetic features, protein expression, etc., which can enable doctors to select matching targeted therapies and targeted drugs for patients. This has made the search for highly effective and low-toxicity targeted drugs a hot topic in current research [7]. Using key enzymes in cellular signal transduction pathways related to tumor cell differentiation and proliferation as drug screening targets, researchers have identified novel drugs with high efficiency, low toxicity and high specificity that selectively act on specific targets.

Protein tyrosine kinases (PTKs) are a group of proteins with tyrosine kinase activity and epidermal growth factor receptor tyrosine kinase (EGFR TK) is a very important receptor tyrosine kinase [8]. The epidermal growth factor receptor is often expressed at high levels in different cancers and its expression levels are positively correlated with cancer progression and poor prognosis [9]. Although research has demonstrated that tyrosine kinase inhibitors have made great advances in the treatment of tumors, tumors are complex diseases characterized by multiple genetic and molecular alterations that affect cell proliferation, survival and differentiation, and tumor growth and survival are not dependent on just one receptor or one signaling pathway [10], making it impossible to kill tumor cells completely by acting on just one target, and tumors have become increasingly resistant to single-target tyrosine kinase inhibitors [11]. Tumor resistance to single-target tyrosine kinase inhibitors has become increasingly apparent. Studies have shown that gefitinib [12], an oral EGFR TK inhibitor, and erlotinib, another small-molecule EGFR TK inhibitor, have comparable clinical efficacy but poor efficacy in patients with EGFR TK resistance mutations [13] and that long-term single use of drugs such as gefitinib has resulted in multiple cases of clinical resistance and associated adverse effects, with T790M point mutations causing 60% of EGFR inhibitor acquired resistance [14]. Studies have shown that inhibition of the kinase activity of cellular mesenchymal epithelial transition factor (c-Met) can restore the sensitivity of EGFR T790M mutant cancer cells to EGFR TK inhibitors, thereby overcoming the problem of drug resistance [15,16]. C-Met is a hepatocyte growth factor (HGF) receptor, which belongs to the tyrosine receptor superfamily and is the expression of the *c-Met* proto-oncogene [17]. Since the crystal structure of c-Met was resolved in 2002, small-molecule inhibitor studies of c-Met tyrosine kinase have been developed more and more [18]. Among them, crizotinib is a dual c-Met/ALK inhibitor that was approved in the FDA for non-small cell lung cancer in August 2011 [19]. AstraZeneca developed pyrazine and its analogues as a c-Met inhibitor. MK-2461 is a highly selective c-Met small-molecule inhibitor, which is now in a phase II clinical study [20]. PF-2341066 and SU11274 also entered clinical research because of their high inhibitory activity and selectivity. Their inhibitory activity on c-Met tyrosine kinase is 5 nM and 10 nM [21], respectively. ARQ-197 acts on the c-Met tyrosine kinase ATP binding site, which is characterized by strong inhibitory activity, good safety and high bioavailability and is now in a phase III clinical trial [22]. It is a product of the *c-Met* proto-oncogene. Recent studies have shown that the combined use of EGFR TK inhibitors and c-Met inhibitors can effectively inhibit the growth of multiple tumor cells significantly better than either alone [23], thus necessitating the design and synthesis of multi-target inhibitors targeting EGFR and c-Met to develop highly effective, low toxicity, multi-target anti-tumor inhibitors.

Based on the pharmacological activities of the above two types of inhibitors, according to their structural characteristics and targets, this study optimized, designed and synthesized a multi-target kinase inhibitor based on an aminopyrimidine skeleton, which can act on both c-Met and EGFR targets to enhance drug affinity and efficacy. We investigated the molecular mechanism of the effect of PYR26 on the proliferation and apoptosis of HepG2 cells and further investigated the effect of PYR26 on the growth of subcutaneous transplanted tumors in nude mice and the growth indexes of nude mice. Accordingly, we can obtain a new generation of multi-target tumor inhibitors with high efficiency, low dose and low toxicity to provide a more effective drug for cancer treatment and to provide a new theoretical basis and research method for rational drug design. At the same time, this study was expected to verify whether such multi-target inhibitors targeting EGFR and c-Met can not only achieve the inhibition of c-Met kinase, but also reduce various side effects caused by the combination of drugs.

## 2. Results

### 2.1. Inhibitory Effect of PYR26 on the Activity of HepG2 Cells

**Figure 1:**
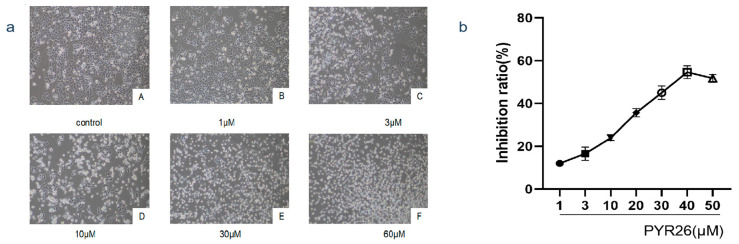
(**a**) Effect of PYR26 on amount of HepG2 cells (×100). The cultures were grown for 24 h, and the medium was changed to that containing various concentrations of PYR26 (final concentration: (**A**) control, (**B**) 1 μΜ, (**C**) 3 μΜ, (**D**) 10 μΜ (**E**) 30 μΜ and (**F**) 60 μΜ) for another 24 h. (**b**) Cell growth survival of CCK-8 assay was measured at 24 h after the cells were exposed to PYR26.

After treating HepG2 cells with different doses of PYR26 for 24 h, comparing with the control group, as the concentration of PYR26 increased, the number and density of cells gradually decreased, and the cell edges gradually became inconspicuous, the cells piled up into clusters, crumpled into circles and broke into fragments as shown in Figure 1a. The inhibition rate of HepG2 cells in the wells with PYR26 concentrations of 1 μΜ, 3 μΜ, 10 μΜ, 30 μΜ and 60 μΜ was calculated to be 12.0%, 16.6%, 23.9%, 45.0% and 72%, respectively, compared to the control group by the cell counting method. When the PYR26 concentration was 1 μM, the decrease in cell number and density was not significant; when the PYR26 concentration was 10 μM, the number and density of HepG2 cells decreased significantly relative to the control group, and cell debris and cells crumpled into round shapes started to appear. When the concentration of PYR26 reached 30 μM, most of the HepG2 cells died, leaving only the apoptotic cells and cell fragments that were crumpled into circles. Therefore, it was tentatively shown that PYR26 could inhibit the proliferation and growth of HepG2 cells.

As shown in Figure 1b, with the increase in PYR26 concentration, the activity of HepG2 cells was significantly reduced in all groups and the growth inhibition rate was significantly increased, indicating that PYR26 has certain cytotoxicity, and with the increase in PYR26 dose, the growth inhibition rate of HepG2 cells gradually increased, with a dose effect, and the difference was significant (*p* < 0.001). The concentration for 50% of maximal effect (EC50) was 21.44 μM.

### 2.2. Effect of PYR26 on the Nuclear Morphology of HepG2 Cells

**Figure 2:**
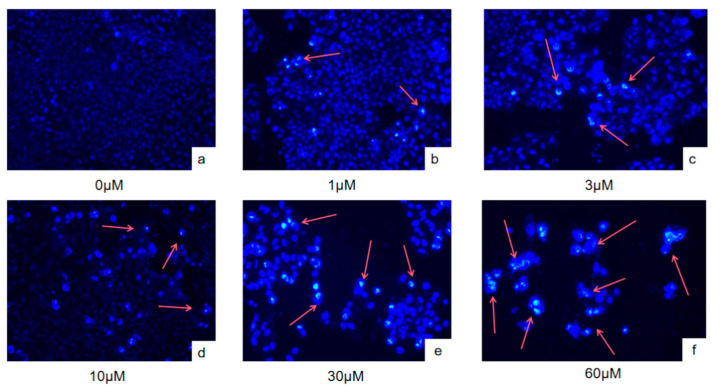
Effect of PYR26 on the morphology of cell nuclei (×400). (**a**) control, (**b**) 1 μΜ, (**c**) 3 μΜ, (**d**) 10 μΜ, (**e**) 30 μΜ and (**f**) 60 μΜ. Red arrows pointed to the bright blue fluorescence emitted by the apoptotic cells.

As shown in Figure 2, the nuclei of the blank control group without PYR26 treatment showed diffuse and uniform fluorescence, which indicated that most of the cells were alive. When HepG2 cells were treated with 30 μΜ PYR26, we could see high staining density, nuclear condensation, cell membrane blistering, nucleolus cleavage, chromosome condensation around the nuclear membrane and apoptotic vesicle formation, all of which were characteristic of programmed apoptosis. As the concentration of PYR26 increased, the apoptotic features of HepG2 cells became more obvious, with a certain dosage effect.

### 2.3. Effect of PYR26 on Reactive Oxygen Species (ROS) Clusters in HepG2 Cells

**Figure 3:**
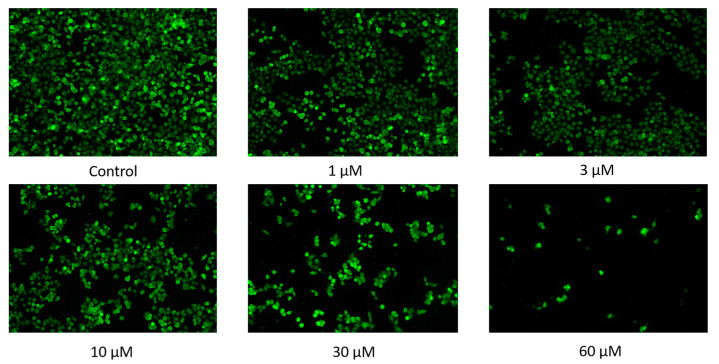
Effect of PYR26 on the ROS of cells.

As shown in Figure 3, the PYR26-induced HepG2 cellular ROS (labeled with DCFH-DA probe) content did not change significantly, compared to the control group, and may not be responsible for the induction of apoptosis in HepG2 cells.

### 2.4. Effect of PYR26 on Apoptosis and on the Cell Cycle of HepG2 Cells

**Figure 4:**
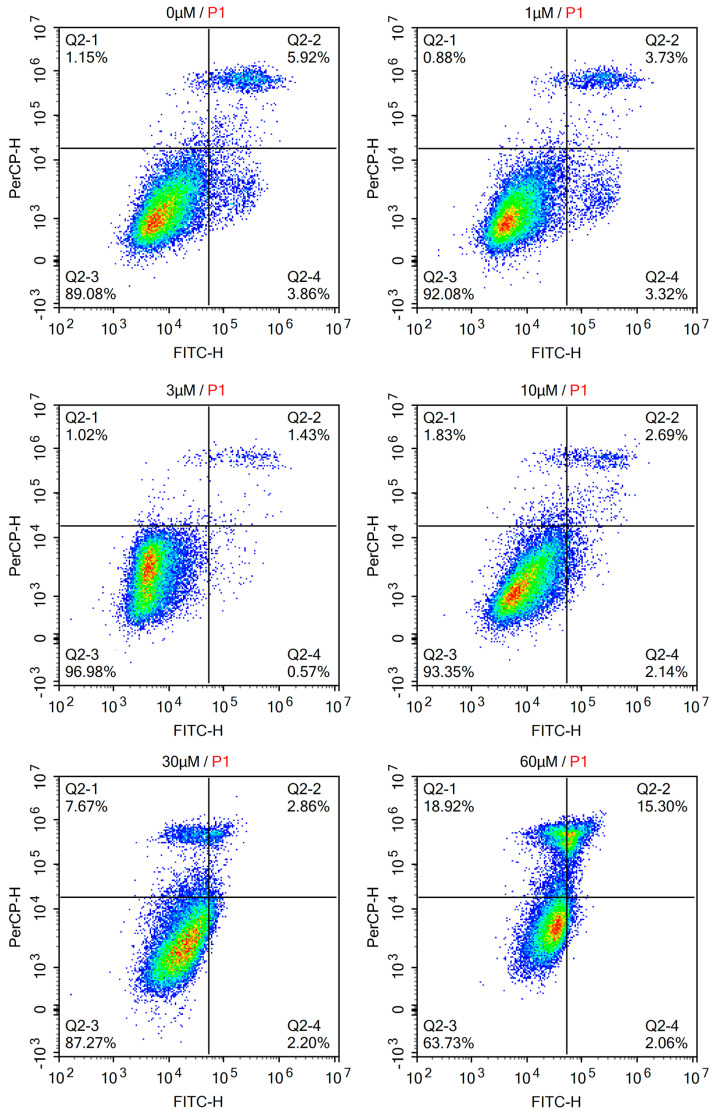
Pro-apoptotic effect of PYR26 on HepG2 cells confirmed by Annexin VFITC/PI. Q2-1 shown necrotic cells, Q2-2 shown late apoptotic cells, Q2-3 shown normal cells, and Q2-4 shown early apoptotic cells.

The pro-apoptotic effect of PYR26 was further confirmed by Annexin VFITC/PI. Compared to the blank control group, the apoptosis rate of the treated cells was increased and HepG2 cells were significantly reduced after 60 µM PYR26 treatment, as shown in Figure 4. The results suggest that PYR26 can promote apoptosis in HepG2 cells.

### 2.5. Effect of PYR26 on CDK4, c-Met, Bak and Caspase-3 Gene Expression in HepG2 Cells

**Figure 5:**
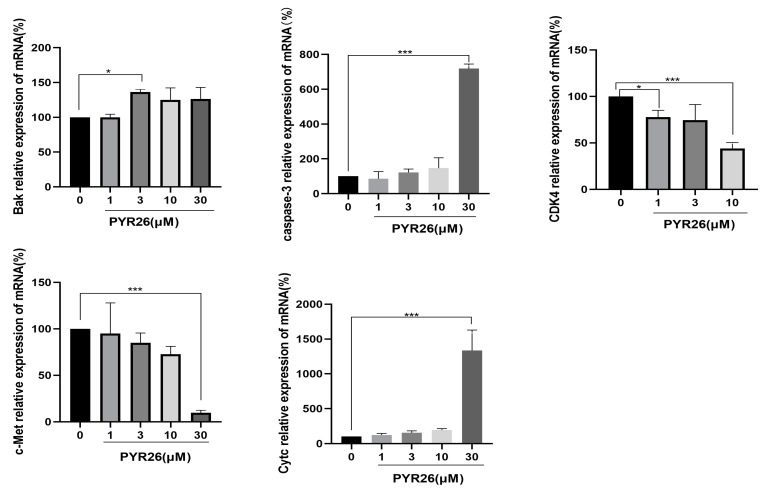
The expression of c-Met, CDK4, Cytc, Bak and caspase-3 genes in HepG2 cells influenced by PYR26 for 24 h (* *p <* 0.05, *** *p* < 0.001 vs. control group).

As shown in Figure 5, after treatment of HepG2 cells with different concentrations of PYR26 for 24 h, the mRNA expression of the *CDK4* gene was lower than that of the control group, in the 3 μM group the expression was significantly lower than that of the blank group (*p* < 0.05) and in the 10 μM group the expression was extremely significantly lower than that of the blank group (*p* < 0.01), with a dose effect. The mRNA expression of the *c-Met* gene was significantly lower in the 30 μM group than that in the control group (*p* < 0.01). It was suggested that PYR26 inhibited cell migration and adhesion by down-regulating the expression of the hepatocyte growth factor receptor (*c-Met*) gene on the surface of HepG2 cells. The expression of *Bak* mRNA was up-regulated in the experimental groups (1 μM, 3 μM, 10 μM, 30 μM) compared with the control group, with the highest up-regulation in the 3 μM experimental group compared with the control group (*p* < 0.05). The results showed that PYR26 could promote apoptosis in HepG2 cells by up-regulating the mRNA expression of *Bak*, a pro-apoptotic gene. Compared with the control group, the expression of *caspase-3* mRNA in the treated groups (1 μM, 3 μM, 10 μM, 30 μM) was higher than that of the control group, indicating that PYR26 could up-regulate and activate the expression of *caspase-3* mRNA. Among them, the change in the 30 μM group was significant, and the difference was statistically significant (*p* < 0.01). Compared with the control group, the expression of *Cytc* mRNA in the experimental groups (1 μM, 3 μM, 10 μM, 30 μM) all indicated an increase, among which the expression in the 30uM experimental group was significantly higher than that in the control group, and the difference was statistically significant (*p* < 0.01). The results indicated that PYR26 could promote the release of cytochrome c (*Cytc*), a key signaling molecule of apoptosis, by up-regulating the upstream pro-apoptotic *Bak* gene, activating the downstream activation of the *caspase-3* gene and promoting apoptosis in HepG2 cells and activating the apoptotic pathway.

### 2.6. Effect of PYR26 on HepG2 Cells’ Caspase-3, PI3K, ERK, CDK4 Protein Expression

**Figure 6:**
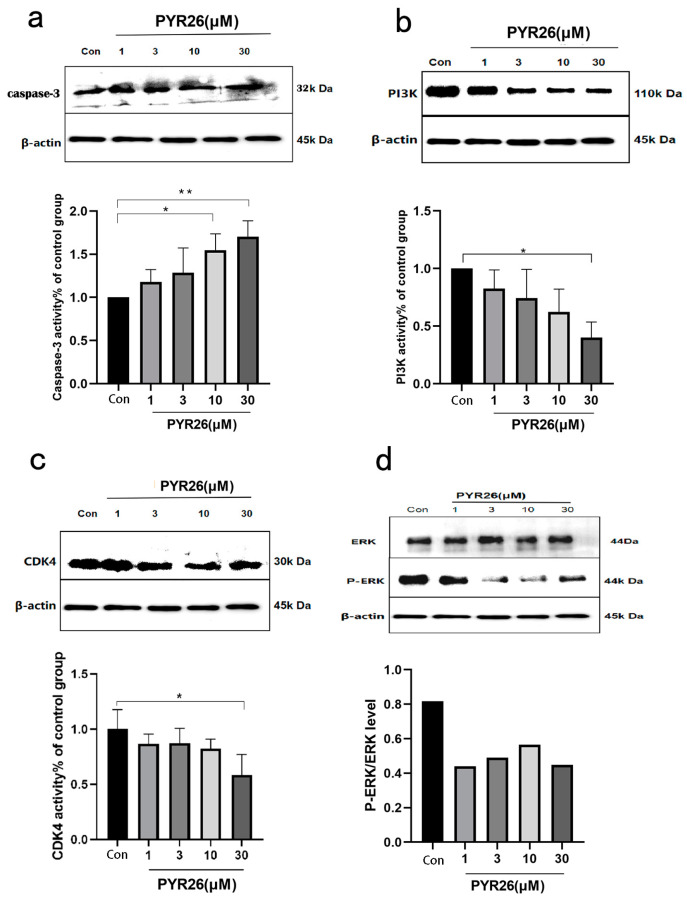
Effect of PYR26 on the expression of caspase-3, PI3K, CDK4 and p-ERK proteins in HepG2 cells after 24 h administration. (**a**) Western blot analysis showed that the levels of caspase-3 were increased by PYR26; (**b**) Western blot analysis showed that the levels of PI3K were inhibited by PYR26; (**c**) Western blot analysis showed that the levels of CDK4 were inhibited by PYR26; (**d**) Western blot analysis showed that the levels of ERK were inhibited by PYR26 (* *p* < 0.05, ** *p* < 0.01 vs. control group).

The results of the experiment are shown in Figure 6. Firstly, the expression of apoptotic factor caspase-3 in HepG2 cells increased, causing the cells to enter programmed death. PYR26 had an inhibitory effect on PI3K protein and CDK4 protein expression with a concentration effect, and PI3K expression decreased, inhibiting the proliferation of HepG2 cells. In addition to this, PYR26 inhibited the expression of ERK protein with a concentration effect. The ERK signaling pathway plays a critical role in cellular physiological processes such as proliferation, survival, differentiation, apoptosis, motility and metabolism.

### 2.7. Effect of PYR26 on Body Index and Tumor-Related Gene Expression in Nude Mice with Liver Cancer

**Figure 7:**
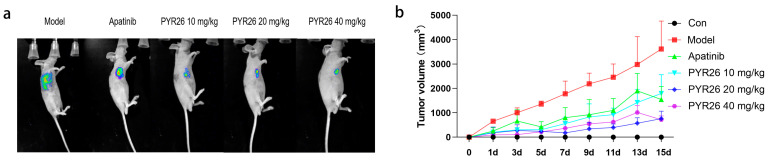
Tumor volume size after drug administration in nude mice. (**a**) Nude mice were given different concentrations of drug 14 d later. (**b**) Curves of tumor volume change at 14 d in nude mice with blank, model, positive control, low PYR26, medium PYR26 and high PYR26.

**Figure 8:**
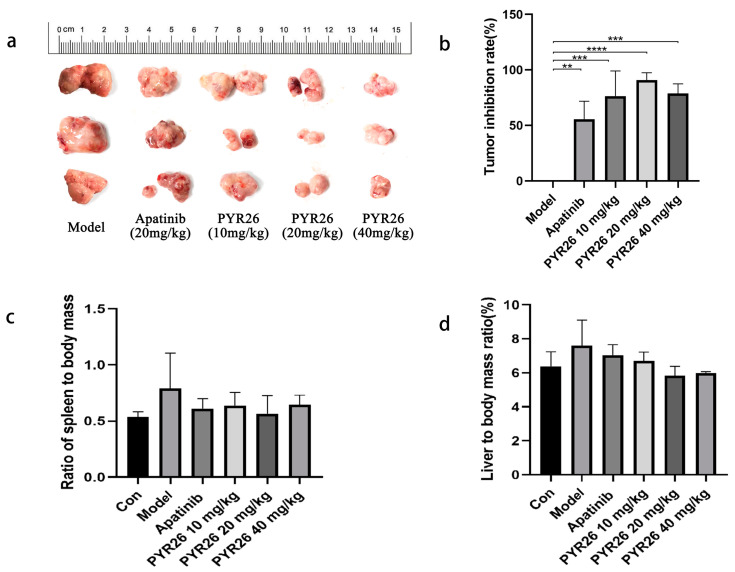
(**a**) Tumors’ size after 14 days of PYR26 administration. (**b**) Tumor suppression rate in mice after 14 days of PYR26 administration. (**c**) Spleen–body ratio of nude mice after 14 days of PYR26 administration. (**d**) Liver–body ratio of nude mice after 14 days of PYR26 administration. (** *p* < 0.01, *** *p* < 0.001,**** *p* < 0.0001 vs. control group).

**Figure 9:**
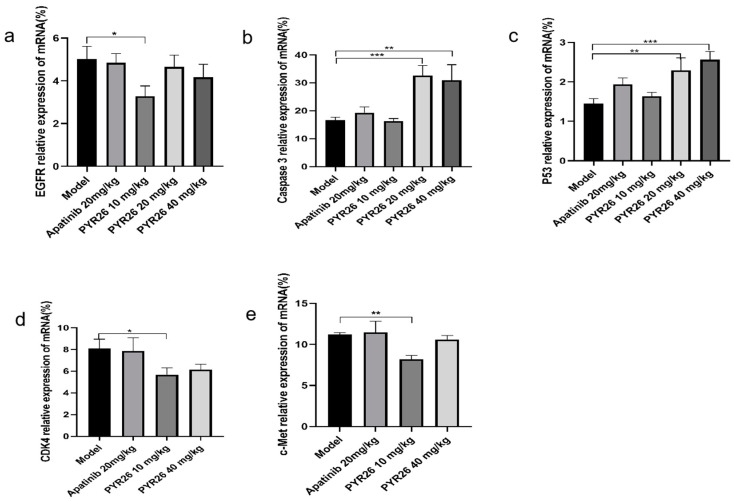
Expression of EGFR, caspase-3, p53, CDK4 and c-Met genes in nude mice after 14 days of PYR26 administration (* *p <* 0.05, ** *p* < 0.01, *** *p* < 0.001 vs. control group).

The tumor mass in the low-, medium- and high-concentration PYR26 groups was significantly lighter and the tumor growth was slower than that in the model and positive control groups, as shown in Figure 7. At the end of the experiment, the tumor volumes in the low-, medium- and high-concentration PYR26 groups were smaller than those in the model group and positive control group, so it was initially shown that PYR26 also had an inhibitory effect on the tumors of Hepa1-6 tumor-bearing mice, as shown in Figure 8a,b. The tumor suppression rate of low, medium and high PYR26 concentrations was reached respectively 50.46%, 80.66% and 74.59%, respectively, thus, PYR26 could significantly inhibit the tumor growth of Hepa1-6 tumor-bearing mice. As shown in Figure 8c,d, the body masses of spleen and liver in the model group were significantly higher than those in the blank group, while the spleen-to-body ratio and liver-to-body ratio in the apatinib group and the low-, medium- and high-concentration PYR26 groups were smaller than those in the model group and close to the spleen-to-body ratio in the blank group, indicating that PYR26 had a protective effect on the liver and spleen of the tumor-bearing nude mice. Figure 9 showed that PYR26 could protect the liver and spleen of the tumor-bearing nude mice by down-regulating *EGFR*, *c-Met* and *CDK4* genes and it up-regulated the expression of *caspase-3* and *p53* genes to inhibit tumor proliferation and promote apoptosis.

**Figure 10:**
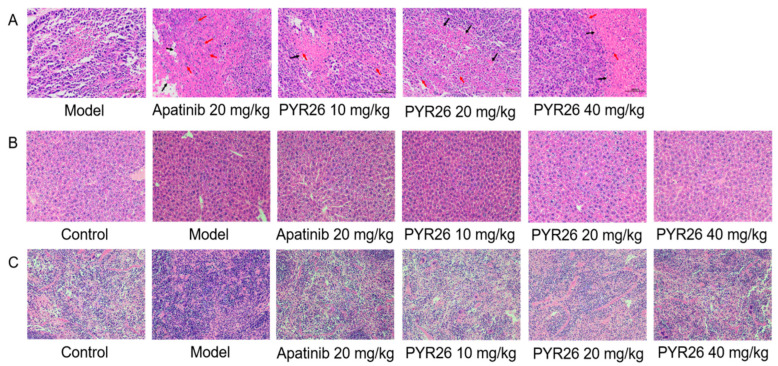
(**A**) In the positive control group (apatinib), the cytoplasmic ratio was high and the nuclei were heteromorphic. About 2/5 of the tumor cells were necrotic in patches, with necrotic nucleolysis and increased eosinophilic cytoplasm (black arrow) and a small amount of granulocyte infiltration in the necrotic lesions (red arrow). The low-concentration PYR26 group showed high cytoplasmic ratio, abnormal nuclei, small focal necrosis regions, nucleolysis, enhanced eosinophilic cytoplasm (black arrow), and small amount of granulocyte infiltration (red arrow). In the medium-concentration PYR26 group, the tumor had high cytoplasmic ratio, abnormal nuclei, focal necrosis of multiple tumor cells, deep staining, dissolution of nucleus pyknosis, enhanced eosinophilic cytoplasm (black arrow), accompanied by a small amount of granulocyte infiltration (red arrow). The high-concentration PYR26 group showed high cytoplasmic ratio and heteromorphic nuclei, with patellar necrosis of nearly half of the tumor cells, lysis of necrotic nuclei, enhanced eosinophilic cytoplasm (black arrow) and a small amount of granulocyte infiltration in the necrotic lesions (red arrow). (**B**) Compared with the control group, model group liver tissue increased cell size and shape, with abnormal, obviously increased nuclear/cytoplasmic ratios, deep staining, disorderly spatial arrangement, with dot necrosis of liver cells and inflammatory cell infiltration. The color of the staining of Apatinib and PYR26 groups is shallow. The hepatic lobule of medium-concentration and high-concentration PYR26 groups are complete, the color becomes light, similar to the blank group. (**C**) In the blank group, the white pulp and red pulp areas of spleen sections were obvious, with obvious demarcation and clear marginal areas, while in the model group, the spleen sections of nude mice had loose texture and no obvious marginal areas. In contrast, the apatinib group and PYR26 group both had obvious marginal areas, and the ratio of red pulp to white pulp was close to that of the blank group (×200).

As shown in Figure 10, the H&E staining results showed that the tumors in both the apatinib and PYR26 groups showed certain necrotic areas. The tumor necrotic areas were larger in the medium-concentration PYR26 group and high-concentration PYR26 group, and the tumor necrosis rate was lower in the low-concentration PYR26 group and apatinib group, indicating that PYR26 has certain anti-tumor activity. Compared to the liver tissue sections of the blank group, the liver cells of the model group were enlarged and abnormal in morphology, with a significantly higher nucleoplasmic ratio, dark staining, disorganized spatial arrangement, punctate necrosis of hepatocytes and inflammatory cell infiltration, while both the apatinib and PYR26 groups had lighter staining, and the liver lobules of the medium- and high-concentration PYR26 groups were intact and significantly lighter in color, similar to the blank group. From the spleen sections of nude mice, the white and red areas of bone marrow were evident in the spleen slices of the blank group. The boundary of the marginal areas was clear. The spleen sections of nude mice in the model group had loose texture and no obvious marginal areas. The PYR26 group had obvious marginal areas, and the ratio of red and white bone marrow was close to that of the blank group. The results above indicated that PYR26 had protective effects on the spleen and could improve the body immunity.

## 3. Discussion

Hepatocellular carcinoma has a high morbidity and mortality rate, with increasing rates, and rapid proliferation, extensive metastasis, high recurrence rate and extremely poor prognosis [24]. At present, the treatment of liver cancer has gradually changed from surgical resection, chemotherapy and radiotherapy [25] to targeted therapy with many advantages such as fewer adverse reactions and high precision [26]. The development of small-molecule targeted anti-cancer drugs with precision targeting, low toxicity and better efficacy has become a new hope for cancer treatment. From 1997 to 2015, the FDA approved 128 anti-neoplastic drugs, and the global anti-neoplastic drug market exceeded USD 100 billion, with targeted drugs accounting for 62% of the total, which have become the mainstream of new anti-neoplastic drugs [27]. The growth and survival of tumors do not only depend on one receptor or one signaling pathway, and the drawbacks such as tumor resistance to single-target inhibitors have become increasingly apparent in clinical studies, while the multi-target inhibitor PYR26 can block both c-Met and EGFR signaling pathways, simplifying the treatment process and promising to achieve a single molecule that both inhibits c-Met kinase and restores EGFR at the same time in T790M mutant cancer cells alongside EGFR TK inhibitors, thus overcoming the problem of drug resistance [28,29].

The novel targeted tumor suppressor (PYR26) was designed and synthesized based on the structural similarities and differences between the crystalline complexes of EGFR and c-Met and their respective ligands, and this study demonstrated the significant proliferation inhibitory effect of PYR26 on HepG2 cells at the cellular level after 24h by CCK-8 and microscopic observation. Hoechst 33258 staining showed that the nuclei of HepG2 cells were crinkled and densely stained, with clustering of chromosome nuclei and an increase in apoptotic vesicles and that PYR26 had a pro-apoptotic effect. In order to further explore more possible mechanisms of apoptosis caused by PYR26, this experiment displayed the effect of PYR26 on the expression of mRNA of *c-Met*, *Bak*, *caspase-3*, *cytochrome c* (*Cyt c*) and other related genes in apoptosis by real-time fluorescence quantitative PCR. It was found that the growth inhibitor could reduce the expression, migration and adhesion ability of *c-Met*, thus achieving the effect of inhibiting hepatocellular carcinoma cells. B lymphocytoma-2 (Bcl-2) is an important proto-oncogene in apoptosis that determines cell survival and death signals, while the Bak gene belongs to a class of pro-apoptotic proteins in the Bcl-2 family. During apoptosis, the pro-apoptosis-related proteins undergo protein processing modifications and insert into the outer membrane of mitochondria, resulting in a decrease in potential, causing the initiation of the release of the pro-apoptotic factor Cytc and the activation of caspase-3, causing apoptosis. PI3K is a major pathway for tumor cell growth and migration and has a pro-proliferative and inhibitory effect on apoptosis, while as an upstream signaling molecule of Bcl-2, the pro-apoptotic protein Bak was confirmed to be reduced in expression under the action of PYR26, and the present study demonstrated by Western blot that PYR26 down-regulated PI3K protein expression significantly. It can be speculated that PYR26 may alter the proliferation of HepG2 cells through the PI3K/Bak/Cytc/caspase-3 pathway and play a role in apoptosis. It has also been shown that CDK4 can bind to survivin to form a complex, releasing substances that bind to caspase-3 and inhibit the activity, which has the effect of preventing apoptosis in hepatocellular carcinoma cells. Cell proliferation is mainly achieved through the regulation of the cell cycle, and the expression and activity of CDK is the core of cell cycle regulation, of which CDK4 is closely related to cell proliferation, affecting the G–S phase of the cell cycle, and its expression level decreases, which was found to play an effect of reducing cell proliferation. From animal experiments, it can be found that PYR26 has a significant inhibitory effect on liver cancer tumor growth, and within a certain concentration range, the higher the concentration of PYR26, the better the cancer inhibitory effect.

## 4. Materials and Methods

### 4.1. Experimental Materials

#### 4.1.1. Experimental Animals

Healthy male SPF-grade BALB/c nude mice, 16–18 g, 4 weeks old, were purchased from the Guangdong Experimental Animal Centre (all experimental operations and animal use were in strict compliance with the “Guideline on the Good Treatment of Laboratory Animals” (Ministry of Science and Technology, China 2006).

#### 4.1.2. Materials and Reagents

Human hepatocellular carcinoma cells, HepG2 cells (the Cell Bank of the Chinese Academy of Sciences, Beijing, China) and Hepa1-6 (the Cell Bank of the Chinese Academy of Sciences), were transfected by Shengbo Biomedical Technology Co. Ltd. (Shanghai, China). Fetal bovine serum (FBS) (Gibco, Logan, UT, USA), double resistance (Gibco), Dulbecco’s modified Eagle medium (DMEM) (Gibco), enhanced Cell Counting Kit-8 (Enhanced CCK-8 kit) (Beyotime Biotechnology, Shanghai, China), Hoechst 33258 (Beyotime Biotechnology), total SOD activity determination kit (Beyotime Biotechnology), reactive oxygen species (ROS) detection kit (Beyotime Biotechnology), hematoxylin and eosin staining reagent (Beyotime Biotechnology), P-ERK, ERK, PI3K, caspase-3, CDK4 and other antibodies (Cell Signaling Technology, Boston, MA, USA), Tranzol Up (Alltech Biotechnology Co. Ltd., Beijing, China), Prime Scrip RT reagent (Beyotime Biotechnology). Scrip^TM^ RT reagent kit with gDNA Eraser, PerfectStart^TM^ Green qPCR SuperMix (TaKaRa, Osaka, Japan), primers (Jin Wei Zhi Biotechnology Co. Ltd., Suzhou, China) were used. All other kits were acquired from Sigma-Aldrich (St. Louis, MO, USA).

#### 4.1.3. Instruments

A CO_2_ incubator (MCO-170AICUVL-PC, Panasonic, Osaka, Japan), microplate reader (Infinite^®^ 200 Pro, Decken Trading Co., Ltd., Shanghai, China), PCR instrument (T100M, Bio-RAD, Shanghai, China), live imager (PET-G4, Rward Life Technology Ltd., Shenzhen, China) and paraffin slicing machine (RM2235, Leica, Germany) were used.

### 4.2. Experimental Methods

#### 4.2.1. Preparation of PYR26, a Multi-Target Kinase Inhibitor Based on the Aminopyrimidine Backbone

Crystal structures of EGFR and c-Met in complex with their respective ligands were first downloaded from the Protein Data Bank website. Then, based on the inhibitor species in the respective crystal complexes, classification and correction were carried out by using the Discovery studio 3.5 software. Then, splitting these small molecule ligands by Schrodinger’s Canvas into fragments containing 2–3 benzene ring structures was carried out, and 3D pharmacophore fingerprint, surface polarization area ratio, torsion energy, potential energy, and electronic potential energy were measured. The active skeleton was screened out. Furthermore, the simulated docking of the active backbone with the active site in the crystal complex of EGFR and c-Met proteins, respectively, was carried out. Finally, a 3D-QSAR model was constructed for molecular simulation calculation and scoring [30,31,32]. Finally, potential multi-target inhibitors based on EGFR and c-Met were screened out.

##### PYR26 Synthesis

(1) Compound 1 (20 mmol) was dissolved in 100 mL of anhydrous isopropanol, then compound 2 (20 mmol) and N, N-diisopropylethylamine (40 mmol) were slowly added and the reaction was followed by TLC for 12 h under reflux conditions. At the end of the reaction, the reaction solution was cooled to room temperature and poured into water (100 mL), then washed three times with ethyl acetate (200 mL) to extract the organic phase, which was then de-watered with anhydrous magnesium sulfate and concentrated to give product 3 by column chromatography.


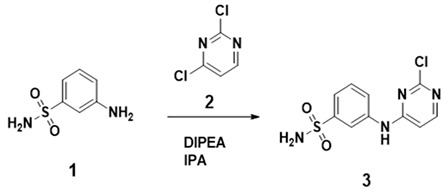


(2) The resulting product 3 (10 mmol) was dissolved in anhydrous ethylene oxide, followed by the sequential addition of boronic acid compound 4 (15 mmol), tetrakis (triphenylphosphine) palladium (1 mmol) and aqueous potassium carbonate (30 mmol). The reaction system was evacuated 3 times and then filled with argon, the reaction solution was heated to 100 °C and the reaction was followed by TLC for 15 h. At the end of the reaction, the reaction solution was cooled to room temperature and poured into water (100 mL), then washed three times with ethyl acetate (200 mL). The organic phase was then removed with anhydrous magnesium sulfate and concentrated to give product 3 by column chromatography. The target compound PYR26 was obtained by column chromatography in 85% yield. NMR data are listed as follows: ^1^H NMR (500 MHz, DMSO-*d*_6_) *δ* 10.12 (s, 1H), 8.75 (t, *J =* 2.0 Hz, 1H), 8.53–8.43 (m, 3H), 8.11 (s, 1H), 8.00 (d, *J* = 8.3 Hz, 2H), 7.78 (dt, *J* = 8.0, 1.6 Hz, 1H), 7.58 (t, *J =* 7.8 Hz, 1H), 7.54–7.42 (m, 2H), 7.39 (s, 2H), 6.81 (d, *J* = 5.9 Hz, 1H).


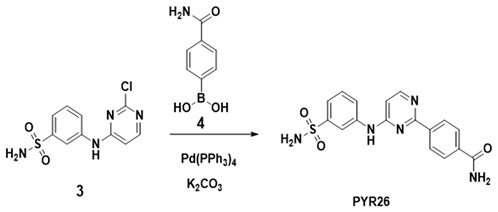


#### 4.2.2. Pharmacological Activity Tests

##### Cell Culture and Processing

HepG2 cells were cultured in DMEM containing 100 mL/L fetal bovine serum, 100 U/mL penicillin and 100 U/mL streptomycin. The flasks were incubated in an incubator at 5% CO_2_ and 37 °C saturated humidity. When HepG2 cells reached 70–80% wall growth, they were digested with 0.25% trypsin for 2 min and divided into six-well plates at a density of 1.0 × 10^5^ cells/mL. Different doses of PYR26 were added to the experimental group (so that the final concentrations were 1 μM, 3 μM, 10 μM, 30 μM and 60 μM, respectively), while the control group received an equal amount of DMEM culture medium. The HepG2 cells were incubated in the incubator for 24 h. The growth of HepG2 cells was observed under an inverted microscope and photographed.

##### CCK-8 Assay for Cellular Activity

HepG2 cells were inoculated in 96-well plates at a density of 5.0 × 10^4^ cells/mL and incubated for 24 h. Different doses of PYR26 were added to the experimental group (so that the final concentrations were 1 μM, 3 μM, 10 μM, 20 μM, 30 μM, 40 μM and 50 μM, respectively), and three replicate wells were incubated for 24 h. After incubation, 10 μL of CCK-8 was added to each well and incubated in the incubator for 1 h. The OD value of each well was measured at 450 nm on an enzyme marker and the experiment was repeated three times. The cell survival and growth inhibition rates were calculated from the OD values. The software GraphPad Prism 8 was used to calculate the concentration for 50% of maximal effect, EC50.

Formula: Cell survival rate = [(As − Ab)/(Ac − Ab)] × 100%;

Cytostatic rate = [(Ac − As)/(Ac − Ab)] × 100%;

where As: experimental wells, Ac: control wells and Ab: blank wells.

##### Morphological Analysis

After 24 h, each well was fixed with 4% paraformaldehyde for 15 min, rinsed with PBS, then stained with Hoechst 33258 staining for 10 min at room temperature, rinsed again with PBS and finally observed under an inverted phase contrast microscope. The cells were finally observed under an inverted phase contrast microscope (200×).

##### Measurement of Amount of Intracellular Activity Oxygen Clusters (ROS) 

HepG2 cells were cultured at an inoculum density of 1 × 10^5^ cells/mL, and cells for the experiment were obtained by adding PYR26 for 24 h according to the previously designed reagent concentration. The amount of reactive oxygen clusters in the cells was determined by fluorescence staining using DCFH-DA. The PYR26-treated HepG2 cells were incubated with 10 mM DCFH-DA for 30 min at 37 °C and washed twice with PBS to obtain the amount of DCF production by enzyme marker reading at 500 nm excitation and 530 nm emission wavelengths.

##### Detection of Apoptosis and Cell Cycle of HepG2 Cells after Drug Administration by Flow Cytometry

HepG2 cells in the logarithmic growth phase were inoculated into 6-well plates and incubated at 37 °C in a 5% CO_2_ incubator for 24 h. PYR26 was added according to the previously designed reagent concentration and incubated continuously for 24 h to obtain the cells required for the experiment. The cells were collected in EP tubes by centrifugation at 1000× *g* for 5 min and the supernatant was discarded. Then, 1 × Binding Buffer was added to resuspend the cells at a concentration of 1.0 × 10^6^ cells/mL. One hundred microliters of the above cell suspension was pipetted into another centrifuge tube, 5 µL of Annexin V-FITC was added in sequence, 5 µL of PI was added and the reaction was carried out at room temperature (25 °C) and protected from light for 15 min. Four hundred µL 1 × Binding Buffer was added to each tube and flow analysis was run using the appropriate channel (FL1 for FITC, FL3 or FL2 for PI).

##### Detection of Cellular Gene Expression by Real-Time Fluorescence Quantitative PCR

Logarithmic growth phase HepG2 cells were taken and inoculated in 6-well plates. After 24 h incubation at 37 °C in a 5% CO_2_ incubator, different concentrations of PYR26 were added to each well according to the previously designed reagent concentration to continue incubation. After 24 h, the culture medium was poured out and washed once with PBS. Total RNA was extracted and 1 μL of each sample was taken for concentration measurement. The remaining samples were stored at −80 °C for long-term use.

Reverse transcription was performed using the PrimeScript™ RT reagent kit with gDNA Eraser (TaKaRa, Osaka, Japan) to uniformly obtain cDNA. PCR reaction solution was prepared on ice and a real-time PCR reaction was performed. Gene expression in HepG2 cells after dosing was measured by pre-denaturing at 95 °C for 30 s, followed by a PCR reaction: 95 °C for 5 s and finally 60 °C for 30–60 s.

##### Western Blot to Detect Protein Expression

Logarithmic growth phase HepG2 cells were taken and inoculated in 6-well plates. After 24 h incubation at 37 °C in a 5% CO_2_ incubator, the culture was continued by dosing according to the previously designed reagent concentration. After 24 h, the plate was placed on ice, the original culture medium was discarded, washed 3 times with PBS, lysed, collected, placed on ice on a shaker for at least 30 min, centrifuged at 13,400× *g*, 4 °C, for 15 min and the supernatant was collected. The protein concentration was determined using the BCA kit (Beyotime Biotechnology, Shanghai, China) to determine the loading volume. A 4×loading buffer was added to the protein sample (V_loading buffer_:V_sample_ = 1:3) and denatured in boiling water at 99 °C for 5 min.

After adding 10 µg of protein per lane, polyacrylamide gel (SDS-PAGE) electrophoresis and 8–12% PAGE electrophoresis, the proteins were transferred to nitrocellulose membranes, washed 5 times with TBST and closed with 5% skimmed milk for 2 h. Then, samples were washed with TBST for 3 times, primary antibody (1:1000) was incubated overnight at 4 °C, washed 3 times with TBST and secondary antibody of sheep anti-rabbit IgG (1:5000) was added and incubated 5 times with TBST for 10 min each. The bands were incubated for 1 h at room temperature on a shaker and washed 5 times for 10 min each time with TBST. The ECL chemiluminescence reagent was added and the bands were developed and photographed on a Bio Light imaging system (Bio-Rad, Hercules, CA, USA).

##### Animal Experiments

(1) Hepa1-6-luc Cell Culture

Hepa1-6 cells were transfected with luc fluorescence and cultured in DMEM containing 100 mL/L fetal bovine serum, 100 μ/mL penicillin and 100 μ/mL streptomycin. Culture flasks were incubated in an incubator at 5% CO_2_ and 37 °C saturated humidity. When Hepa1-6-luc cells reached the logarithmic growth stage, the culture medium was replaced with fresh culture medium and cells were collected the next day.

(2) Establishment of a transplantation tumor model for liver cancer in nude mice

Eighteen 4-week-old male SPF-grade BALB/C nude mice were prepared and housed in an SPF environment at 26–28 °C with a relative humidity of 30–40% and a 12 h/12 h light/dark cycle with free access to food and water. Modeling was carried out when the nude mice had been acclimatized for one week and had a body mass of 18–20 g. We randomly selected sixteen nude mice for modeling. The remaining three served as blank controls. The cells were collected, centrifuged at 4 °C and 160× *g*, the supernatant was discarded, resuspended in PBS, 6 μL was taken for cell counting, centrifuged again, the supernatant was discarded, resuspended in 0.9% physiological saline injection and an equal volume of matrix gel was added. The cell suspension at a concentration of 1 × 10^7^ cells/mL was injected subcutaneously into the right axilla of nude mice at a concentration of 0.2 mL each.

(3) Drug Administration

Tumor formation was observed once every 2 days after modeling. When the tumors grew to 50–200 mm^3^, in vivo imaging was performed and the nude mice were divided into 6 groups based on the principle of equal tumor volume. There were three nude mice in each group. These included the high-concentration group (PYR26 40 mg/(kg-dose)), the medium-concentration group (PYR26 20 mg/(kg-dose)), the low-concentration group (PYR26 10 mg/(kg-dose)), the positive control group (apatinib 20 mg/kg) and the model group, which was fed the same volume of normal saline. After 7 d, the tumors were imaged in vivo once every two days, the body weight of the nude mice was measured every two days and the length and diameter of the tumors were measured with vernier calipers. The tumor volume was calculated according to the following formula:

V = ab^2^, (tumor length: a, tumor diameter: b).

(4) Sampling and Preservation

The nude mice were weighed on day 15 of administration and anesthetized with 350 mg/kg chloral hydrate. Immediately after anesthesia, the nude mice were fixed on a foam board with a needle and perfused with 0.9% physiological saline to fill the perfusion tube, the infusion set was inserted into the left ventricle and the needle was slowly inserted upwards into the aorta for body circulation. The tumor tissue was removed intact, except for necrotic sites and vascular enriched sites, the liver and spleen were taken, washed with PBS, aspirated, weighed and group photographed and tumor volume and in situ wet weight of these organs were recorded. Measured tumor suppression rate, tumor volume suppression rate and organ index were calculated using the following formula.

tumor inhibition rate (%) = [(mean tumor mass of model group − mean tumor mass of drug group)/mean tumor mass of model group] × 100%

tumor volume inhibition rate (%) = [(mean tumor volume in model group − mean tumor volume in dosing group)/mean tumor volume in model group] × 100%

Visceral index = visceral mass/body mass.

(5) QPCR for Gene Expression

The supernatant was collected, added with the same volume of isopropanol as RNAiso Plus and left to stand for 10 min at room temperature, then centrifuged at 13,400× *g*, 4 °C for 15 min. The supernatant was washed with 75% ethanol and then centrifuged at 13,400× *g* for 5 min at 4 °C. The supernatant was discarded, and the precipitate was dried and dissolved in an appropriate amount of DEPC water for measurement of RNA concentration. Reverse transcription and QPCR assays were performed as in Section 4.2.2.

(6) H&E Staining

Tumor, liver and spleen tissues were dehydrated, embedded and made into paraffin sections, which were dewaxed, hydrated and stained in hematoxylin solution and eosin stain, dehydrated in gradient alcohol and sealed in xylene. The sections were observed with an inverted phase contrast microscope and photographed.

#### 4.2.3. Statistical Analysis

All data are shown as mean ± standard deviation. Statistical analysis was one-way analysis of variance with GraphPad for significance of differences between multiple groups of samples. The criterion for all significance in the test was *p* < 0.05.

## 5. Conclusions

In summary, the study demonstrates that the novel targeted tumor suppressor (PYR26) inhibits the growth of HepG2 cells and Hepa1-6 tumors. Meanwhile, tumorigenesis is the result of multi-pathway interactions, and PYR26 has significant effects on the expression of several related proteins and mRNAs in the molecular mechanisms of cell proliferation and apoptosis, further revealing the regulatory roles and mechanisms of related target genes, providing new targets and strategies for clinical drug development of and tumor treatment with small-molecule tumor inhibitors, as well as for the screening of other types of multi-target inhibitors. It also provides new ideas and methods for the screening of other types of multi-target inhibitors.
